# Introduction of a Novel Structure for a Light Unmanned Free Balloon’s Payload: A Comprehensive Hybrid Study

**DOI:** 10.3390/s24103182

**Published:** 2024-05-17

**Authors:** Norbert Hegyi, Gusztáv Fekete, János Jósvai

**Affiliations:** 1 Department of Vehicle Production and Technology, Audi Hungaria Faculty of Automotive Engineering, Széchenyi István University, H-9026 Győr, Hungary; josvai@sze.hu; 2 Department of Material Science and Technology, Audi Hungaria Faculty of Automotive Engineering, Széchenyi István University, H-9026 Győr, Hungary

**Keywords:** novel payload structure, dodecahedron, finite element method, PLA, low air pressure test, drop test, flight test

## Abstract

Payloads for light unmanned free balloons must meet several safety requirements such as being able to protect the inner electronics in order to extract scientific data and to reduce the chance of inflicting personal injury in case of an accidental fall. This article proposes a novel payload structure, which exhibits the form of a dodecahedron. The actual form was determined by carrying out theoretical drop tests on different polyhedrons using the finite element method (FEM). From the simulations, it could be deduced that the dodecahedron was the optimal choice, since the duration of the impact was longer, while the impact force was slightly lower. The payload was produced by additive technologies; therefore, after performing tensile tests on probable materials, PLA was selected as the optimal candidate. The theoretical results about the dodecahedron’s ability were validated by laboratory and real-life drop tests, where the new payload was subjected to 56% less impact force under a 78% longer collision time compared to a classic, rectangular cuboid design. Based on these tests, it was demonstrated that the new structure is safer and it is applicable.

## 1. Introduction

The Commission Implementing Regulation (EU) No. 923/2012 regulates unmanned free balloons in the European Union. In the regulation, three categories are defined: light, medium and heavy unmanned free balloons. The light unmanned free balloons have a payload of one or more packages with a combined mass of less than 4 kg [1]. Light unmanned free balloons usually consist of three main parts: a balloon, a parachute and a payload. In some special designs, the parachute is not part of the instalment [2,3]. The payload itself is often a radiosonde.

In the development of light unmanned balloons, which serve mostly scientific purposes, one major point is safety. A malfunction, namely the fall of a balloon, can, on the one hand, cause directly hazardous situations and even lethal accidents to humans and animals [4,5], while, on the other hand, the complete destruction of the payload can lead to the loss of scientific data. An accidental fall of the balloon must be prevented or at least the damage should be controlled in a way that it would not lead to personal injury or data loss.

To obtain a safer payload package, several aspects needed to be taken into consideration, such as the geometrical structures and the used materials. In order to make an optimal decision about the geometric form of the outer shell, the inside frame and the applied material, the most probable choice analysis and experiments had to be carried out.

The payload of balloons can be formed from various geometric objects. A geometric object with a high number of faces, edges and vertices has a greater chance of survival for stronger collisions since the impact force can be distributed in different directions.

Several geometric objects are available; therefore, a thorough analysis was needed to choose the optimal one. Platonic solids (tetrahedron, cube, octahedron, dodecahedron and icosahedron), a cuboid, a cylinder and a sphere were the reviewed options. Their characteristics are described on which their pro and contra features can be distinguished [6,7].

It must also be mentioned that the geometry of a usual payload is often a rectangular box with sharp edges. This feature can cause serious damage or injury upon collision [4]; therefore, our research aims to provide a possible form or forms that not only secure the survival of the payload but also prevent personal injury.

The payload of balloons usually has relatively high stiffness, which is mainly credited to their outer structure, formed from special, often polyurethane (PUR), foams. It has been demonstrated that foams under compressive impact loading exhibit higher energy absorption compared to non-foam-type materials [8]. Mechanical properties such as strain rate and strain effects of PUR foams have evolved in recent decades, so they were the primary options for a novel partial foam packaging [9,10].

The inner structure, or frame, has multiple roles; therefore, several needs must be satisfied. The frame must be able to support the outer shell; it also must be capable of securing itself and the inner electronic parts inside. It must have the attribute of deforming or even falling apart upon collision to reduce the emerging impact force. In addition, radio waves must be able to emit through the structure. Moreover, it is also an important goal that the production of the inner frame and the unique structural parts could be carried out in a cheap and simple way. Consequently, 3D additive manufacturing should be considered to create such a frame system [11].

With regard to additive manufacturing, primary supported materials must be chosen that meet certain conditions, such as user-friendliness, easy printing characteristics, light density, optimal strength, good hardness, non-toxicity and non-flammable, and last but not least, it must let radio waves pass through.

When the possible additive materials are considered, it can be concluded that carbon fibre composite and copper composite filaments contain radio wave insulating materials [12], which do not allow them to be used for this purpose [13]. If wood composites are considered, whose properties are always combined with their binding polymers, usually polylactic acid (PLA), then this material could be applicable. All the same, their price is higher than several other normal additive materials like acrylonitrile butadiene styrene (ABS), thermoplastic polyurethane (TPU) or PLA.

If the TPU material is considered, it must be noted that this material is too flexible (it would act like a spring, causing additional damage during collision) and it is more expensive than normal ABS or PLA materials. ABS has a relatively high strength, a relatively high impact resistance and also a higher toughness (e.g., Fillamentum Industrial ABS Extrafill or extrudr’s DuroABS filament). ABS materials have a shorter elongation at break compared to TPU but longer compared to PLA (e.g., Fillamentum Industrial PLA Extrafill filament or extrudr PLA NX2 filament). Again, ABS is usually more expensive than PLA. If PLA is considered, it should be regarded that it is less impact-resistant and has the shortest elongation at break compared to ABS and TPU.

As for production, ABS requires higher temperatures for printing compared to PLA. ABS needs to have a heated print bed usually between 80 and 110 °C. To improve layer adhesion and also to reduce warping, an enclosed workspace is required. PLA can be printed usually on a printing bed with a temperature between 15 and 70 °C. PLA is considered a cheap material; moreover, it is a biodegradable thermoplastic, mostly produced from renewable biological resources, so it has less impact on the environment compared to ABS. Based on the pros and contras of the above-mentioned materials, the possibilities can be narrowed down to ABS and PLA as the optimal choices.

Based on the relevant references, two main objectives were set for this paper.

As for the first objective, a new geometric inner structure should be established by means of finite element methods for light unmanned free balloons’ payloads, which lacks sharp edges to avoid personal injury but exhibits the ability of being subjected to the lowest impact force possible. The second characteristic ensures a higher possibility of survivorship. Our second objective is to find an adequate additive material for the new inner structure, which secures the survivorship of the payload under collision. This objective is to be carried out by experimental tests carried out on possible materials.

To support the validity and applicability of the new inner structure together with the chosen material, a low air pressure test, laboratory drop test and real-life flight test were performed.

## 2. Materials and Methods

### 2.1. Selection of Optimal Inner Geometry Structure

Polyhedrons were considered in this paper as possible and applicable geometrical forms since a geometry with a high number of faces, edges and vertices can distribute the impact force in different directions. Table 1 summarises the force-leading properties in a weighted rating form for polyhedrons. It must be noted that, through these variables, it becomes possible to pre-evaluate the theoretical force distribution ability of these polyhedrons. The rating breaks down each property into weighted values. The best is 1, and 0 is unusable. The options were calculated with Equation (1) to obtain the ratio of the compared number to look for the best option.

(1) $$Force\;ratio\;(Fr)=\frac{compared\;number}{highest\;number}$$

As seen in Table 1, the dodecahedron, icosahedron and sphere have identical results. Consequently, these are the possibilities that should be reduced to one object.

The sphere is made of 12 outside-rounded pentagons, which is almost the same as the dodecahedron. However, the rounded forms lead to more material in the layer compared to a regular dodecahedron’s flat surface. In addition, if the sphere was a hided icosahedron, it would also be heavier compared to a regular icosahedron. A bigger mass would lead to a heavier payload, which should be avoided. Although a sphere provides smaller air resistance (or smaller drag coefficient), it shows no significant differences during low-velocity flights, based on our experience.

A regular dodecahedron or a regular icosahedron could also be a possible option.

A Platonic icosahedron, with the same inradius as a dodecahedron, has 9.8% less volume than a dodecahedron. A larger volume is better to place the needed inner payload module (e.g., electronic parts). In addition, a larger volume can provide more space for deformation and lead, presumably, to a softer collision. Nevertheless, the dodecahedron’s outer shell mass is also 9.8% bigger than an icosahedron’s. As another aspect, an icosahedron’s single face is also smaller than a dodecahedron’s, which may lead to a higher energy density upon collision for a smaller area.

To be able to choose between a dodecahedron and an icosahedron, a finite element method (FEM) simulation has been carried out to view a virtual mechanical collision, which also has shock characteristics. FEM modelling methods for structural damage detection and collision can be used under 10 m/s impact speeds, which is the same region of values in this study [14,15].

**Figure 1:**
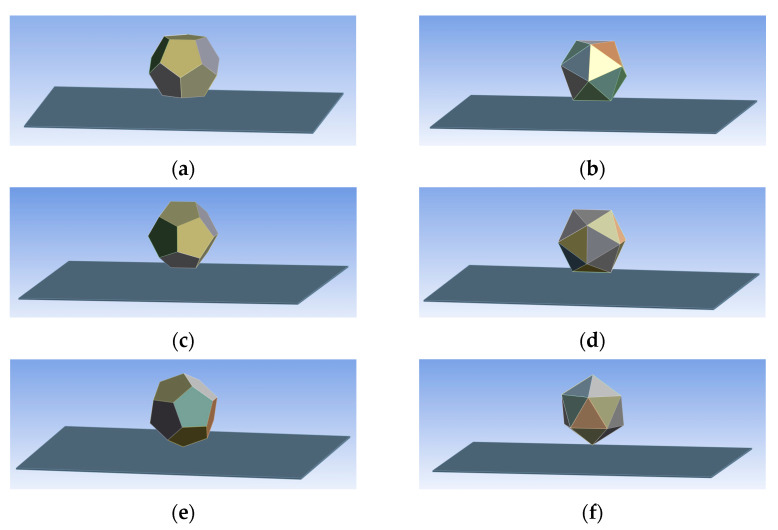
FEM simulation of drop test: (**a**) dodecahedron “face-to-face” collision and (**b**) icosahedron “face-to-face” collision; (**c**) dodecahedron “edge-to-face” collision and (**d**) icosahedron “edge-to-face” collision; (**e**) dodecahedron “vertex-to-face” collision and (**f**) icosahedron “vertex-to-face” collision.

As previously mentioned, the inradius of each geometrical object is an important parameter for the supporting volume to the inner electronic parts, etc. In the simulations, the dodecahedron and the icosahedron had the same inradius. The thickness of the walls was 10 mm, and the inradius of the objects was 100 mm. The equal inradius resulted in different masses between the geometrical objects. The length of the edge of an icosahedron was 132.3 mm, while the edge of a dodecahedron was 89.8 mm. The icosahedron object’s summarised edges were 47.4% longer than those of the dodecahedron object. This meant that the frame for these parts was heavier. Six simulations were performed with the software Ansys Workbench 16.2, where the object was defined as a low-flow polypropylene (PP) shell, while the landing area was defined as an aluminium plate. All versions were virtually dropped from a 50 mm height with an initial speed of 10 m/s, leading to a 10.12 m/s collision speed to a supported 1000 mm × 1000 mm × 5 mm plate. Explicit physical preference was chosen, with a linear element order. The meshing had cuboid elements with 10 mm sizes. The dodecahedron version’s meshing had 11,284 elements with 23,538 nodes, while the icosahedron version’s meshing had 11,324 elements with 23,882 nodes. Both geometrical forms were dropped as “face to face”, ”edge to face” and “vertex to face” in Figure 1.

In Figure 1a,b, the parallel face-to-face collision can be seen when one of the body’s faces collided parallel to the plate’s centre. In Figure 1c,d, the edge-to-face collision is shown when one edge of a body collides parallel to the surface of the plate. In Figure 1e,f, one vertex of the body collides with the plate surface’s centre. Upon collision, the duration and force need to be compared. The maximal force persisted for a fraction of a millisecond (0.4–0.5 ms). In Table 2, the duration of collision, the maximal force and the average forces were compared.

Based on the above-mentioned parameters (duration, maximal and average forces), we can conclude that the dodecahedron performs slightly better; therefore, it was chosen for further application.

### 2.2. Selection and Testing of Optimal Materials for Inner Geometry Structure

As a first step to select the optimal material for the pieces of the dodecahedron’s frame, Fillamentum ABS Extrafill Metallic Grey, Fillamentum PLA Extrafill Traffic White filament and two versions of ecoPLA filament were purchased from 3DJake (Paldau, Austria).

To make a decision about which material should be used in order to build a new frame, tensile and compression tests were chosen to find clear differences between the properties of ABS and PLA materials. This decision was based on the proposal of the relevant literature [16,17].

Before the mechanical tests, the printability of the materials was examined.

A sample piece was designed in Creo 6.0 CAD software, for testing purposes, with the following properties: 43.5 mm long square section, with a 5.5 mm side dimension and a 4 mm diameter ball joint at the end. The piece also had a 44 mm × 35 mm × 5.5 mm sized rectangular cuboid part as a fixation for tensile and compression testing. The diameter of the ball joint connection was chosen to be printable but as small as possible. The parts for testing were printed with a 0.1 mm layer height, a 0.4 mm shell thickness and a 0.5 mm top–bottom distance. The shell thickness is set to the minimum (0.4 mm), which is the diameter of the basic nozzle.

Fillamentum ABS Extrafill Metallic Grey, two versions of 3DJake ecoPLA filament and the Fillamentum PLA Extrafill Traffic White filament were printed with the Anycubic 4 Max 2.0 machine. The ecoPLA did not provide stable quality as some pieces had failures in shell and layer stability and surface quality. The pieces printed from Fillamentum ABS Extrafill Metallic Grey and Fillamentum PLA Extrafill Traffic White had much better quality, and no failures in the shell or layer instability were observed; therefore, they were chosen as the materials.

In order to maintain reproducibility, the parts were always printed in the same printing directions, with the same speeds, temperatures, layer heights, shell thicknesses, top–bottom thicknesses and ball joint connections. Two parts were snapped together mechanically to form a ball joint connection.

It must be mentioned that the ball joints could not only snap together but also apart. This option was chosen because ball joints have a limited range of movement in all directions, which is necessary for the deformation of the frame and outer shell at the moment of collision.

Tensile and compression tests were carried out on the ABS and PLA material parts at the Széchenyi István University’s Material Testing Laboratory with an Instron 5588 universal testing machine. For each material, 5 samples were made with a 4 mm length. For each test, the maximal tension (T) force and maximal compression (C) force were measured, as shown in Table 3.

The difference between the ABS and PLA parts can be clearly seen in Table 3. At the ABS ball joint connections, the average tensile force is only 1–7% higher, while the compression force is 2.4–2.6 times higher compared to PLA. A weaker connection and a weaker compression force are better for this purpose. Therefore, PLA was chosen as the material.

As for the soft outer shell of the payload, an open-cell flexible polyurethane foam (PUR foam) has been chosen with soft, flexible, heat-insulating properties, described in our earlier work [18].

### 2.3. Design and Production of Inner Geometry Structure

As payloads can have relatively high stiffness, the absorbing frame structure must be able to reduce damage during collision. For parts produced with fused deposition modelling (FDM), there are several methods to make them more resilient without modifying the used material. One example is the use of inner supporting structures in the printed parts [19].

The basic idea was to construct so-called “vertexes” for a frame structure to hold the dodecahedron’s formed outer shell together. To create such a structure, Creo Parametric computer-aided design (CAD) software was used. The vertex parts have ball joints with the ability to carry out a limited range of smooth deformation in all directions. The parts and their connections had to be strong enough to hold the whole structure with the mass of the shell and the inner electronic parts.

The inner frame pieces were designed in CAD software. The parts were produced with an Anycubic 4Max Pro 2.0 FDM machine using PLA material. The vertex parts had 3 arms separated at an angle of 120°. These arms were 45 mm long, and they stretched from the middle vertical axis to the midpoint of the ball joint connection.

The diameter of the ball joints was set to 4 mm. The layer height of all parts was 0.1 mm, with a single-line 0.4 mm shell thickness and 0.5 mm top–bottom height.

**Figure 2:**
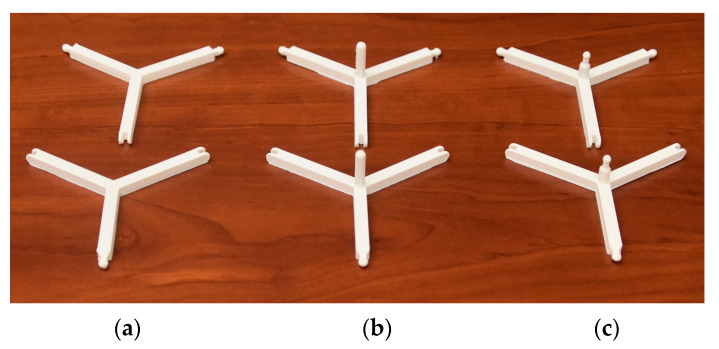
The 3D-printed normal frame parts (**a**); parts with outstanding pillars (**b**); parts with outstanding pillars and a sphere for a ball joint (**c**).

In Figure 2, the printed vertex parts are shown. In Figure 2a, the frame parts without any protrusion components are visible. These are the normal frame parts. In Figure 2b, the parts with protrusion components or pillars are shown. These pillars support the inner payload (e.g., electronics) from the downside as later described. In Figure 2c, the parts have protrusion pillars, with a sphere for the ball joint. These parts have a ball joint connection with a pentagonal ring downside as later described.

**Figure 3:**
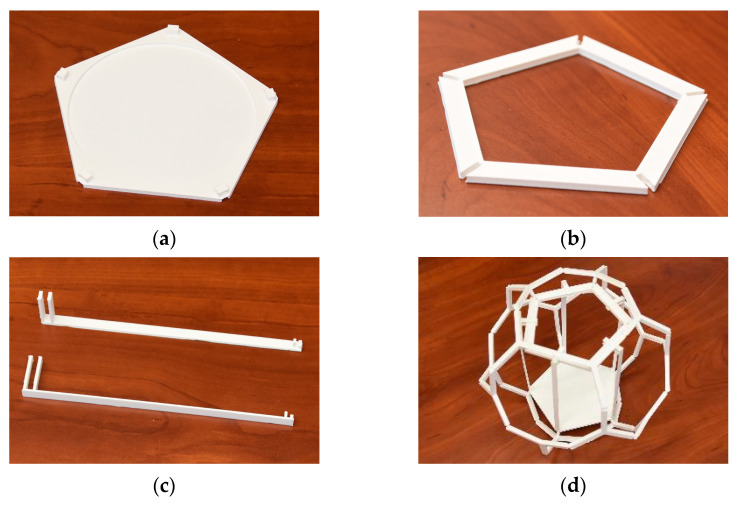
The 3D-printed pentagonal ring (**a**), supporting part (**b**), fixation for possible inner payload (**c**) and the complete model (**d**).

The fixation to the downside of the inner parts (e.g., electronic inner payload) is made by a flat part, as shown in Figure 3a. The previously mentioned pentagonal ring is shown in Figure 3b. This pentagonal ring is fixed with a ball joint to the frame. It also fixes the inner payload with the help of long supporting parts, as shown in Figure 3c.

After all the needed parts were manufactured, the whole frame was built together (Figure 3d). The frame looks like a truncated polyhedron, but after adding the previously mentioned outside PUR foam shell, it becomes a regular dodecahedron.

### 2.4. Low Air Pressure Test

Two low air pressure tests were carried out to detect possible deformation. These tests were performed by expanding the air inside the parts. One test was carried out only with the parts of the frame structure and one with the complete new model.

Both tests were performed with an air pressure simulating machine in the Packaging Laboratory at the Széchenyi István University. The tested pieces were placed inside the machine on an A3-sized piece of paper with 10 × 10 mm sized black and white squares. A camera and a lamp were placed above them. The camera took photos every 5 s.

**Figure 4:**
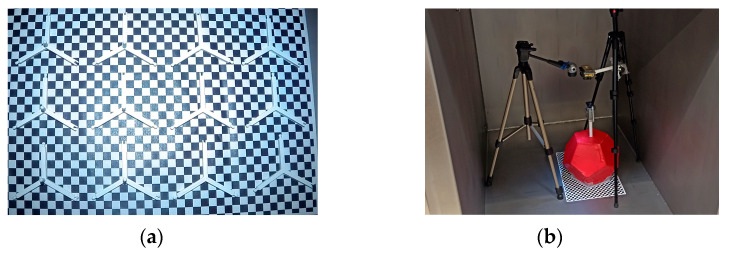
The 3D-printed parts (**a**) and the whole dodecahedron formed structure (**b**) inside the low air pressure chamber.

The purpose of the first test was to reveal if the parts of the frame structure would expand at low air pressure. As shown in Figure 4a, 12 samples (6 normal parts, 6 parts with pillars) were placed inside the machine. The temperature was set to 20 °C at the beginning of the test. The test began at a pressure of 1003 hPa, which gradually reduced until 80 hPa was reached (which is equal to the air pressure at a 17.6 km altitude) in 24 min. None of the samples showed any deformation.

The purpose of the second test was to reveal if the whole new model would expand at low air pressure. As shown on the right side of Figure 4b, one model was placed inside the machine. The test began at a pressure of 1001 hPa, and 80 hPa was reached in 24 min.

Similarly, the new, complete model did not show any deformation either.

### 2.5. Laboratory Drop Test

After the low air pressure test, a drop test was carried out with the purpose of measuring the collision force of the two different payloads (the dodecahedron design and the rectangular cuboid design). In the case of drop tests, it is worth mentioning that shock damage to a package has 3 main factors: the peak acceleration, the change in velocity and the duration of the whole process.

In this drop test, the measured force and the duration were the investigated factors. To obtain the needed information about the collision process, the Savitzky–Golay filtering method and RStudio software (version number 2023.03.0-daily+82.pro2 with R programming language) were used to process the data [20]. Video recordings were also employed for the drop tests, 3 times in each case. Recordings were taken at a rate of 2000 fps and 1280 × 1024 pixel resolution with an iX i-SPEED 3 high-speed camera with a Tamron AF 28–300 mm f/3.5–6.3 XR Di LD Aspherical Macro objective. This video system allowed for the visual detection of deformations in 0.5 ms time steps.

The test was performed at the Packaging Laboratory, where the payloads (the dodecahedron and classic cuboid box) were dropped from a 4.8 m height onto a 20 mm × 800 mm × 520 mm sized (height × length × width) steel plate. Impact forces were measured for each case five times by pressure cells with a maximum accuracy of 0.05%.

The box shell had dimensions of 134 ± 2 mm × 234 ± 2 mm × 185 ± 1 mm (height × length × width), a wall thickness of 17.5 ± 0.5 mm, a mass of 285–305 g and a final mass of 880–900 g. The box shell was made of expanded polystyrene (EPS).

The biggest surface area (the downside) was the colliding face. The new dodecahedron shell had an edge length of a = 100 mm, a circumsphere radius R = 140.1 mm and a wall thickness of 21 mm. The circumsphere radius can be calculated by the following formula, where “a” is the length of edge [21]:

(2) $$R=a\cdot \frac{1}{4}\cdot \left(\sqrt{15}+\sqrt{3}\right)$$

The final mass of the dodecahedron body was 1100–1200 g, and it was made of soft Smooth-On FlexFoam-iT! III Polyurethane foam (PUR foam). Both of the payloads had an impact speed of 8.6 m/s.

It needs to be mentioned that both the box and dodecahedron package were dropped with an exactly balanced mass centre, so the slight differences upon impact were most probably caused by other factors, such as the opening of the gripper. It can be hypothesised that the gripper did not open at exactly the same time, and it led to a small twisting motion, which increased during the fall. It needs to be stated that real-life atmospheric conditions, together with the balloon and the parachute, will always cause the payload packages to swing and twist like a pendulum before the moment of impact. Consequently, this means that the laboratory drop test demonstrated a close-to-real-life situation.

**Figure 5:**
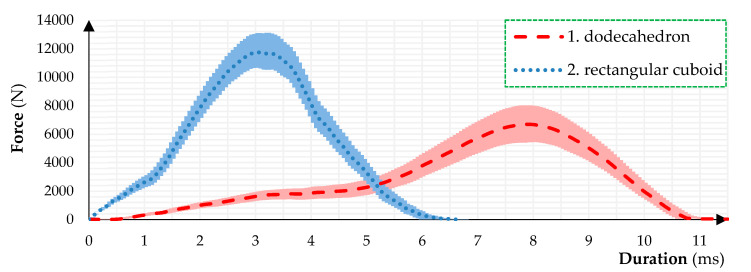
Force and duration of collisions: 1st graph drop test of a dodecahedron; 2nd graph drop test of a rectangular cuboid.

In Figure 5, two graphs can be seen. The 1st line (continuous line) shows the force evolution during impact with regard to the dodecahedron payload. The 2nd line (dashed line) demonstrates how the force progresses during impact when the rectangular cuboid payload is considered. It can be clearly seen that the rectangular cuboid’s collision results exhibit not only a higher force but a shorter duration as well compared to the dodecahedron’s collision.

Table 4 shows the processed measured data of the separate drop tests. By inspecting the average of the maximal forces, it could be concluded that the dodecahedron payload package was subjected to 56% less impact force under a 78% longer collision time. This means that even though a heavier dodecahedron structure has higher impulse and pressure, due to its softness, it would result in less damage compared to the more rigid rectangular cuboids (with EPS material) since it transmits less impact force to the target with the same inner payload mass.

**Figure 6:**
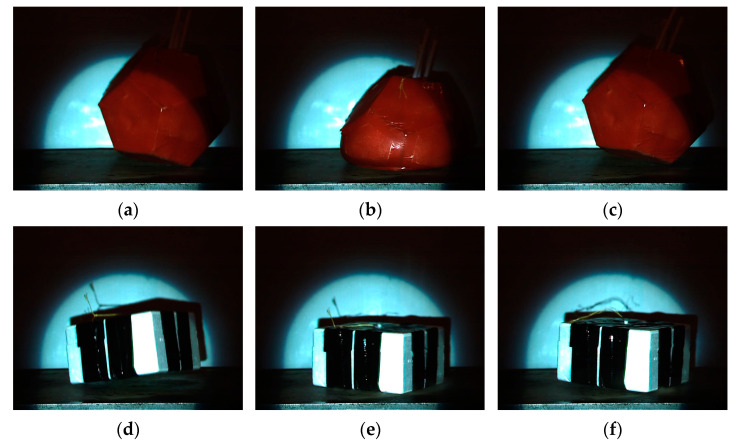
High-speed video frames at collision with two types of payload package: (**a**) a dodecahedron’s first contact with target; (**b**) a dodecahedron’s maximal deformation; (**c**) a dodecahedron bouncing back; (**d**) a rectangular cuboid’s first contact with target; (**e**) a rectangular cuboid’s full contact with target surface; (**f**) the rectangular cuboid bouncing back.

By analysing the frames, it can be stated that all parts of the collision can be separately seen and differentiated. Figure 6a shows the dodecahedron payload’s first contact with the target plate. In Figure 6b, the maximal deformation of the dodecahedron payload is shown. Figure 6c shows the dodecahedron payload leaving the target plate. In Figure 6d, a rectangular cuboid payload’s first contact with the target is shown. In Figure 6e, the full contact with the target surface is shown. Figure 6f shows the rectangular cuboid leaving the target plate.

**Figure 7:**
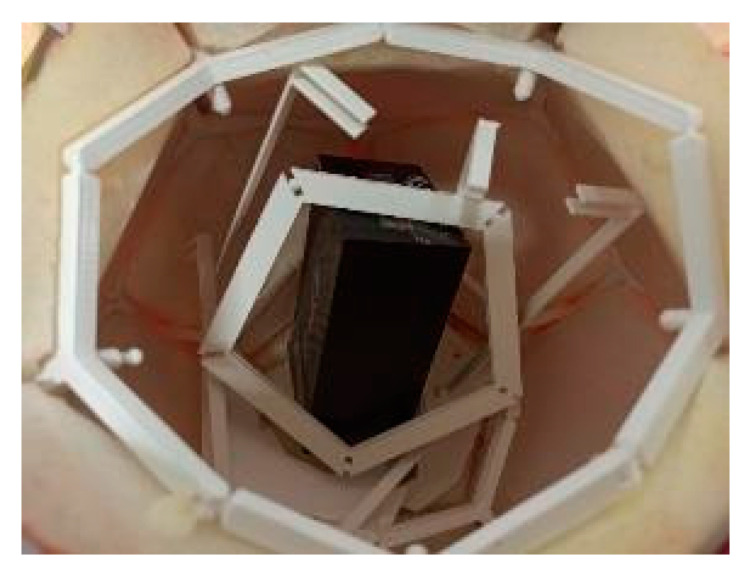
Payload’s inside after drop test.

In Figure 7, the inside of a payload with the extra weight can be seen after a drop test. The energy absorption of a movable and also breakable frame structure with its soft outer layer absorbs more impact energy; therefore, it potentially deforms more. The force of impact is distributed inside a larger area and over a longer duration, which reduces the peak force.

### 2.6. Real-Life Flight Test

The first real-life flight test was carried out on 30 October 2021 with the new dodecahedron payload. The duration of the flight lasted for 1 h 37 min. The test started at Hegyhát Observatory in Hegyhátsál (Hungary), whilst its landing site was at Potyon (Hungary). The maximum reached altitude was 27,465 m, with a top speed of 293 km/h, achieved at 25,378 m altitude. The mass of the payload was 1.6 kg, whereas its descent speed was 2.8 m/s in the last minute before touchdown.

Its primary tracking was performed with 3 reprogrammed Vaisala RS-41-SG radiosondes for 3 frequencies in APRS tracking (432.500 MHz, 432.775 MHz, 432.900 MHz). For APRS-tracking HA1NX, the official radio amateur call sign of N. Hegyi was used. Tracking data were transmitted every 60 s on each frequency. During the flight, inside temperature data were also transmitted by the tracking system, which registered a minimal temperature of 4 °C on one side of the payload and 10 °C on the other side of the payload. The payload’s inner downside flat plate was modified to keep the instruments in place for flight tracking. The modified part was planned to be able to fall apart in the vertical direction upon impact with the ground.

**Figure 8:**
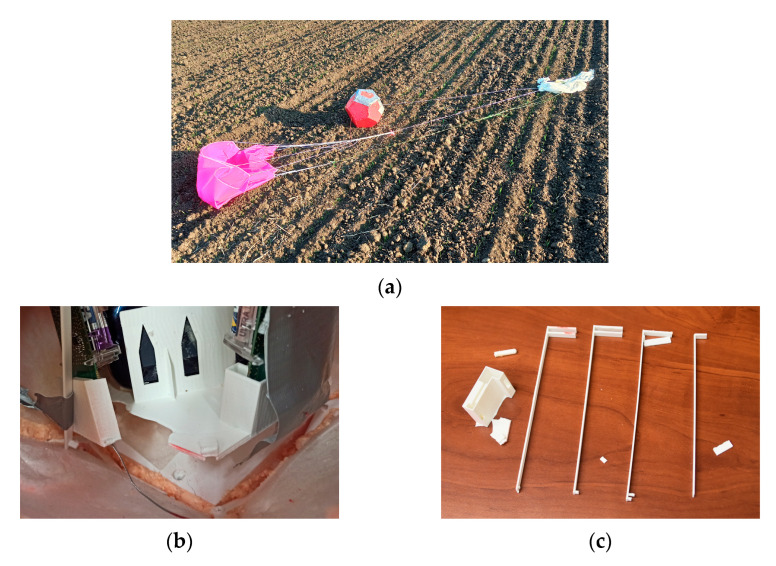
After first flight: after touchdown (**a**), inside (**b**), supporting parts (**c**).

The payload survived the extreme conditions, such as high wind speed or low air pressure. Figure 8a shows the payload after touchdown. Figure 8b demonstrates the bottom part, which is bent and broken as planned. In Figure 8c, the broken supporting inside parts are shown.

## 3. Conclusions and Summary

The development and testing have led to a new, usable payload form, namely the dodecahedron, which has been proven to be a safer structure for light unmanned free balloons. It has also been deduced that the optimal material for this novel payload should be made of PLA. The form and the material were selected following a rigorous analysis and experimental tests.

The above-mentioned results were concluded in several steps.

First, the selection of a geometric design was implemented through the analysis of eight possible geometric forms by means of finite element simulations. The simulations clarified that the dodecahedron form is the most suitable option for a new type of payload if light unmanned free balloons are considered. This choice was made on the basis that the dodecahedron form yielded the lowest impact forces.

Second, the selection of materials was conducted on the basis of tensile tests where two possible materials were examined, PLA and ABS. Since the purpose of the inner frame was to absorb impact energy by carrying out plastic deformation and partial disintegration, PLA was preferred over ABS due to its lower compression abilities.

When the geometrical form and the material were selected, several complete models were manufactured with additive technologies. The production was followed by drop tests, where it was deduced that the newly introduced dodecahedron form could significantly elongate the impact duration (78% more), which also resulted in a lower (56% less) impact force. The high-speed camera tests validated the existence of the elongated plastic deformation when the dodecahedron–PLA geometry–material combination was applied.

As final proof of the new payload’s performance, a real-life flight test and post hoc visual analysis were carried out where it was proven that the model could protect the inner electronics.

## Figures and Tables

**Table 1:** Summarised properties for comparison.

Geometrical Object	Force (from Face to Face)	F_r_	Force (from Face to Edge)	F_r_	Force (from Vertex to Faces and Edges)	F_r_	Mean Value
tetrahedron	4	0.2	6	0.20	4	0.2	0.20
cube	4	0.2	12	0.40	8	0.4	0.33
cuboid	4	0.2	12	0.40	8	0.4	0.33
octahedron	8	0.4	12	0.40	6	0.3	0.37
dodecahedron	12	0.6	30	1.00	20	1	0.87
icosahedron	20	1.0	30	1.00	12	0.6	0.87
cylinder	3	0.2	2	0.07	2	0.1	0.11
sphere (12 parts)	12	0.6	30	1.00	20	1	0.87

**Table 2:** Comparison between FEM-generated data.

	Duration (ms)	Maximal Force (N)	Average Force (N)
Dodecahedron face to face	8.7	32,909	14,489.98
Icosahedron face to face	8.6	33,255	14,624.81
Dodecahedron edge to face	9.7	35,016	12,529.55
Icosahedron edge to face	9.4	34,491	14,338.37
Dodecahedron vertex to face	9.4	35,446	14,264.87
Icosahedron vertex to face	7.6	33,880	17,080.75

**Table 3:** Comparison between ABS and PLA parts’ measurements.

Sample	T (1) (N)	C (1) (N)	T (2) (N)	C (2) (N)	T (3) (N)	C (3) (N)
ABS1	6.06	−1.94	5.51	−1.87	5.48	−1.95
ABS2	6.90	−2.53	6.74	−2.23	6.66	−2.40
ABS3	6.96	−2.34	6.41	−2.18	6.09	−2.03
ABS4	6.50	−1.83	5.43	−2.16	5.28	−1.71
ABS5	6.53	−2.25	6.44	−1.96	5.78	−1.52
average	6.59	−2.18	6.11	−2.08	5.86	−1.92
SD	0.36	0.29	0.59	0.15	0.54	0.33
PLA1	6.15	−0.87	5.90	−0.93	5.16	−0.71
PLA2	6.56	−0.87	6.54	−0.98	6.14	−0.89
PLA3	6.25	−0.79	6.01	−0.85	5.50	−0.80
PLA4	6.18	−0.61	5.64	−0.62	5.42	−0.62
PLA5	6.68	−1.36	6.16	−0.56	5.08	−0.81
average	6.36	−0.90	6.05	−0.79	5.46	−0.77
SD	0.24	0.27	0.33	0.18	0.41	0.1

**Table 4:** Comparison between dodecahedron and rectangular cuboid object’s measurements.

Test	Maximal Force (N)	Duration (ms)
Dodecahedron 1	8029.6	11.0
Dodecahedron 2	6109.9	10.8
Dodecahedron 3	7343.1	10.1
Dodecahedron 4	6541.9	9.9
Dodecahedron 5	5414.4	10.9
Average	6687.8	10.5
SD	1025.5	0.5
Rectangular cuboid 1	10,609.2	5.7
Rectangular cuboid 2	11,760.2	6.2
Rectangular cuboid 3	12,523.2	5.8
Rectangular cuboid 4	10,660.5	6.4
Rectangular cuboid 5	13,193.3	5.8
Average	11,749.3	5.9
SD	1136.8	0.3

## Data Availability

Data are contained within the article.
